# 1-(2,4-Dinitro­phen­yl)-3,3-dinitro­azetidine

**DOI:** 10.1107/S1600536809049861

**Published:** 2009-11-25

**Authors:** Biao Yan, Hai-Xia Ma, Yin Hu, Yu-Lei Guan, Ji-Rong Song

**Affiliations:** a School of Chemistry and Chemical Engineering, Yulin University, Yulin 719000 Shaanxi, People’s Republic of China; b School of Chemical Engineering, Northwest University, Xi’an 710069 Shaanxi, People’s Republic of China

## Abstract

In the title compound, C_9_H_7_N_5_O_8_, the dihedral angle between the mean plane of the azetidine ring and that of the benzene ring is 26.1 (1)°; the planes of the two nitro groups of the azetidine ring are aligned at 88.7 (1)°.

## Related literature

Highly nitrated small-ring heterocycles are good candidates for energetic materials because of the increased performance from the additional energy release upon opening of the strained ring system during decomposition, see: Frumkin *et al.* (1999 ▶). Azetidine-based explosives, such as 1,3,3-trinitro­azetidine (TNAZ) demonstrate excellent performance, see: Archibald *et al.*, (1990 ▶); Hiskey & Coburn (1994*a* ▶,*b* ▶). The title compound is a derivative of 3,3-dinitro­azetidine (DNAZ) (Hiskey *et al.*, 1992 ▶, 1993 ▶), which is a derivative of TNAZ. For the use of DNAZ in the preparation of a variety of solid energetic compounds, see: Ma *et al.* (2009*a* ▶,*b* ▶,*c* ▶); Gao *et al.* (2009 ▶).
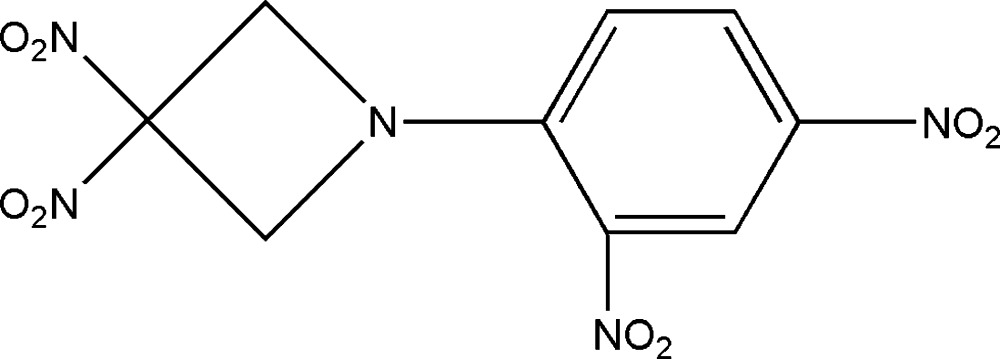


## Experimental

### 

#### Crystal data

C_9_H_7_N_5_O_8_
*M* *_r_* = 313.20
Monoclinic, 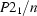

*a* = 8.113 (2) Å
*b* = 10.676 (3) Å
*c* = 14.398 (4) Å
β = 104.681 (4)°
*V* = 1206.3 (6) Å^3^
*Z* = 4
Mo *K*α radiation
μ = 0.15 mm^−1^
*T* = 293 K
0.31 × 0.26 × 0.20 mm

#### Data collection

Bruker SMART APEX diffractometer
Absorption correction: none
5860 measured reflections
2140 independent reflections
1670 reflections with *I* > 2σ(*I*)
*R*_int_ = 0.019

#### Refinement

*R*[*F* ^2^ > 2σ(*F* ^2^)] = 0.038
*wR*(*F* ^2^) = 0.118
*S* = 1.20
2140 reflections
199 parameters
H-atom parameters constrained
Δρ_max_ = 0.21 e Å^−3^
Δρ_min_ = −0.21 e Å^−3^

### 

Data collection: *SMART* (Bruker, 2003 ▶); cell refinement: *SAINT* (Bruker, 2003 ▶); data reduction: *SAINT*; program(s) used to solve structure: *SHELXS97* (Sheldrick, 2008 ▶); program(s) used to refine structure: *SHELXL97* (Sheldrick, 2008 ▶); molecular graphics: *SHELXTL* (Sheldrick, 2008 ▶); software used to prepare material for publication: *SHELXTL*.

## Supplementary Material

Crystal structure: contains datablocks I, global. DOI: 10.1107/S1600536809049861/ng2691sup1.cif

Structure factors: contains datablocks I. DOI: 10.1107/S1600536809049861/ng2691Isup2.hkl

Additional supplementary materials:  crystallographic information; 3D view; checkCIF report

## Figures and Tables

**Fig. 1.**
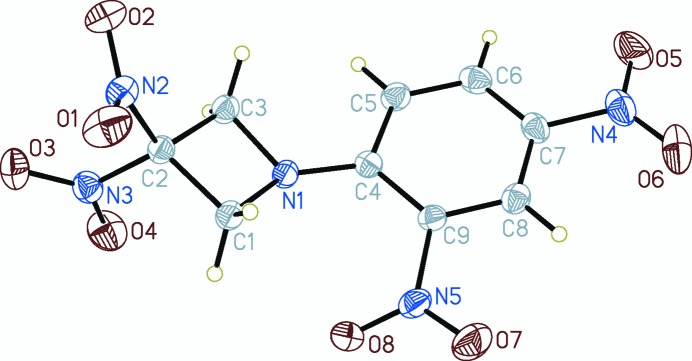
The molecular structure of (I), showing the atom-numbering scheme. Displacement ellipsoids are drawn at the 30% probability level and H atoms are drawn as spheres of arbitrary radius.

## supplementary crystallographic information

### Comment

Highly nitrated small-ring heterocycles are good candidates for energetic materials because of the increased performance from the additional energy release upon opening of the strained ring system during decomposition (Frumkin *et al.*, 1999). Azetidine-based explosives, such as 1,3,3-trinitroazetidine (TNAZ) (Archibald *et al.*, 1990; Hiskey *et al.*, 1994*a*,*b*) demonstrate excellent performance partly because of the high strain associated with the four-membered ring. As one of the important derivates of TNAZ, 3,3-dinitroazetidine (DNAZ) (Hiskey *et al.*, 1992; Hiskey *et al.*, 1993) can prepare a variety of solid energetic compounds (Ma *et al.*, 2009*a*,*b*,*c*; Gao *et al.*, 2009). The title compound (I) is a DNAZ derivates. The dihedral angle between the azetidine ring and benzene ring is 26.1° and the planes of two nitryl of azetidine ring is 88.7°. There are no important intermolecular contacts in the crystal structure.

### Experimental

A solution of DNAZ (0.2353 g, 1.6 mmol), 2,4-dinitrochlorobenzene (0.33 ml, 1.6 mmol), and NaHCO_3_ (0.28 g, 3.2 mmol) in dichloromethane (30.0 ml) was stirred under reflux for 20 h. The reaction mixture was concentrated in vacuo, water (30 ml) was added, and the unstable mixture was extracted rapidly with dichloromethane (3 * 15 ml). The combined extracts were dried (MgSO_4_,), the solvent was concentrated *in vacuo*, and ethanol (20 ml) was added, and the residue was filtrated to give the yellow compound in 30% yield. Crystals were obtained from dichloromethane, by slow evaporation at room temperature. Elemental analysis calculated for C_9_H_7_N_5_O_8_: C 34.61, N 22.36, H 2.253%; found: C 34.61, N 22.22, H 2.249%. IR (KBr, cm^-1^): 3100.29, 1585.18, 1526.85, 1335.18, 1304.76, 869.25, 820.72.

### Refinement

H atoms were placed at calculated idealized positions and refined using a riding model, with C—H distances in the range 0.93–0.97 Å.

### Crystal data

**Table:**

C_9_H_7_N_5_O_8_	*F*(000) = 640
*M**_r_* = 313.20	*D*_x_ = 1.724 Mg m^−^^3^
Monoclinic, *P*2_1_/*n*	Mo *K*α radiation, λ = 0.71073 Å
Hall symbol: -P 2yn	Cell parameters from 1904 reflections
*a* = 8.113 (2) Å	θ = 2.4–26.0°
*b* = 10.676 (3) Å	µ = 0.15 mm^−^^1^
*c* = 14.398 (4) Å	*T* = 293 K
β = 104.681 (4)°	Block, yellow
*V* = 1206.3 (6) Å^3^	0.31 × 0.26 × 0.20 mm
*Z* = 4	

### Data collection

**Table:**

Bruker APEXII diffractometer	1670 reflections with *I* > 2σ(*I*)
Radiation source: fine-focus sealed tube	*R*_int_ = 0.019
graphite	θ_max_ = 25.1°, θ_min_ = 2.4°
φ and ω scans	*h* = −9→9
5860 measured reflections	*k* = −11→12
2140 independent reflections	*l* = −17→17

### Refinement

**Table:**

Refinement on *F*^2^	Primary atom site location: structure-invariant direct methods
Least-squares matrix: full	Secondary atom site location: difference Fourier map
*R*[*F*^2^ > 2σ(*F*^2^)] = 0.038	Hydrogen site location: inferred from neighbouring sites
*wR*(*F*^2^) = 0.118	H-atom parameters constrained
*S* = 1.20	*w* = 1/[σ^2^(*F*_o_^2^) + (0.063*P*)^2^ + 0.0921*P*] where *P* = (*F*_o_^2^ + 2*F*_c_^2^)/3
2140 reflections	(Δ/σ)_max_ = 0.028
199 parameters	Δρ_max_ = 0.21 e Å^−^^3^
0 restraints	Δρ_min_ = −0.21 e Å^−^^3^

### Special details

**Table:**

Geometry. All e.s.d.'s (except the e.s.d. in the dihedral angle between two l.s. planes) are estimated using the full covariance matrix. The cell e.s.d.'s are taken into account individually in the estimation of e.s.d.'s in distances, angles and torsion angles; correlations between e.s.d.'s in cell parameters are only used when they are defined by crystal symmetry. An approximate (isotropic) treatment of cell e.s.d.'s is used for estimating e.s.d.'s involving l.s. planes.
Refinement. Refinement of *F*^2^ against ALL reflections. The weighted *R*-factor *wR* and goodness of fit *S* are based on *F*^2^, conventional *R*-factors *R* are based on *F*, with *F* set to zero for negative *F*^2^. The threshold expression of *F*^2^ > σ(*F*^2^) is used only for calculating *R*-factors(gt) *etc*. and is not relevant to the choice of reflections for refinement. *R*-factors based on *F*^2^ are statistically about twice as large as those based on *F*, and *R*- factors based on ALL data will be even larger.

### Fractional atomic coordinates and isotropic or equivalent isotropic displacement parameters (Å^2^)

**Table:**

	*x*	*y*	*z*	*U*_iso_*/*U*_eq_	
O2	0.56725 (18)	0.14099 (15)	0.95776 (10)	0.0576 (4)	
N1	0.06769 (18)	0.16834 (14)	0.89752 (10)	0.0360 (4)	
C4	−0.0303 (2)	0.25527 (16)	0.92696 (11)	0.0324 (4)	
O8	−0.18514 (19)	0.22095 (15)	0.72839 (10)	0.0566 (4)	
N2	0.4618 (2)	0.14859 (15)	0.88135 (11)	0.0419 (4)	
C9	−0.1501 (2)	0.33688 (17)	0.86745 (11)	0.0334 (4)	
N3	0.3038 (2)	−0.04265 (16)	0.84420 (11)	0.0435 (4)	
N5	−0.20374 (19)	0.32296 (17)	0.76316 (11)	0.0429 (4)	
C1	0.1375 (2)	0.16202 (18)	0.81278 (12)	0.0376 (4)	
H1A	0.1660	0.2430	0.7906	0.045*	
H1B	0.0689	0.1131	0.7602	0.045*	
C3	0.2168 (2)	0.10713 (18)	0.96128 (12)	0.0384 (4)	
H3B	0.1908	0.0288	0.9886	0.046*	
H3A	0.2834	0.1618	1.0105	0.046*	
N4	−0.2936 (2)	0.53426 (18)	1.04331 (15)	0.0556 (5)	
C7	−0.2070 (2)	0.43720 (19)	1.00353 (13)	0.0411 (5)	
O3	0.4390 (2)	−0.07976 (16)	0.83333 (12)	0.0679 (5)	
C6	−0.0999 (2)	0.35558 (19)	1.06406 (13)	0.0441 (5)	
H6	−0.0866	0.3606	1.1300	0.053*	
C2	0.2905 (2)	0.09108 (16)	0.87430 (12)	0.0340 (4)	
C8	−0.2340 (2)	0.42800 (18)	0.90514 (13)	0.0388 (4)	
H8	−0.3082	0.4828	0.8648	0.047*	
O1	0.4830 (2)	0.19814 (17)	0.80972 (11)	0.0655 (5)	
O7	−0.2721 (2)	0.41195 (17)	0.71504 (11)	0.0671 (5)	
O6	−0.3804 (2)	0.61021 (15)	0.98923 (15)	0.0718 (5)	
O4	0.1762 (2)	−0.10369 (15)	0.83337 (12)	0.0678 (5)	
C5	−0.0131 (2)	0.26685 (19)	1.02664 (12)	0.0407 (5)	
H5	0.0596	0.2125	1.0683	0.049*	
O5	−0.2740 (3)	0.5369 (2)	1.13090 (13)	0.0877 (7)	

### Atomic displacement parameters (Å^2^)

**Table:**

	*U*^11^	*U*^22^	*U*^33^	*U*^12^	*U*^13^	*U*^23^
O2	0.0396 (8)	0.0687 (11)	0.0560 (9)	−0.0018 (7)	−0.0034 (7)	0.0035 (7)
N1	0.0336 (8)	0.0430 (9)	0.0315 (7)	0.0082 (7)	0.0083 (6)	0.0019 (6)
C4	0.0289 (9)	0.0349 (10)	0.0344 (9)	−0.0039 (7)	0.0098 (7)	−0.0011 (7)
O8	0.0486 (9)	0.0709 (11)	0.0446 (8)	0.0096 (8)	0.0012 (6)	−0.0167 (7)
N2	0.0366 (9)	0.0427 (9)	0.0457 (9)	0.0007 (7)	0.0092 (7)	0.0018 (7)
C9	0.0295 (9)	0.0389 (10)	0.0311 (8)	−0.0015 (8)	0.0062 (7)	−0.0005 (7)
N3	0.0490 (10)	0.0384 (9)	0.0433 (9)	0.0025 (8)	0.0123 (7)	−0.0007 (7)
N5	0.0306 (8)	0.0574 (11)	0.0387 (9)	0.0037 (8)	0.0050 (7)	0.0027 (8)
C1	0.0342 (10)	0.0451 (11)	0.0338 (9)	0.0067 (8)	0.0089 (7)	0.0017 (8)
C3	0.0375 (10)	0.0404 (11)	0.0364 (9)	0.0059 (8)	0.0075 (7)	0.0039 (8)
N4	0.0443 (10)	0.0497 (12)	0.0779 (13)	−0.0058 (9)	0.0253 (9)	−0.0222 (10)
C7	0.0352 (10)	0.0414 (11)	0.0497 (11)	−0.0052 (8)	0.0164 (8)	−0.0131 (9)
O3	0.0609 (10)	0.0625 (11)	0.0811 (11)	0.0189 (8)	0.0196 (8)	−0.0179 (8)
C6	0.0430 (11)	0.0556 (13)	0.0356 (9)	−0.0058 (9)	0.0135 (8)	−0.0069 (9)
C2	0.0320 (9)	0.0330 (10)	0.0359 (9)	0.0019 (7)	0.0068 (7)	0.0003 (7)
C8	0.0309 (9)	0.0364 (10)	0.0483 (10)	−0.0013 (8)	0.0086 (8)	0.0015 (8)
O1	0.0474 (9)	0.0908 (13)	0.0596 (9)	−0.0145 (8)	0.0159 (7)	0.0171 (8)
O7	0.0700 (11)	0.0777 (12)	0.0478 (8)	0.0224 (9)	0.0043 (7)	0.0165 (8)
O6	0.0689 (11)	0.0460 (10)	0.1116 (14)	0.0094 (9)	0.0432 (10)	−0.0049 (10)
O4	0.0725 (11)	0.0498 (10)	0.0845 (11)	−0.0217 (8)	0.0263 (9)	−0.0118 (8)
C5	0.0397 (10)	0.0480 (12)	0.0339 (10)	0.0030 (9)	0.0083 (8)	0.0032 (8)
O5	0.0877 (13)	0.1094 (16)	0.0693 (11)	0.0103 (11)	0.0260 (10)	−0.0450 (11)

### Geometric parameters (Å, °)

**Table:**

O2—N2	1.2133 (19)	C1—C2	1.530 (2)
N1—C4	1.358 (2)	C1—H1A	0.9700
N1—C1	1.471 (2)	C1—H1B	0.9700
N1—C3	1.473 (2)	C3—C2	1.528 (3)
C4—C5	1.412 (2)	C3—H3B	0.9700
C4—C9	1.420 (2)	C3—H3A	0.9700
O8—N5	1.224 (2)	N4—O6	1.217 (2)
N2—O1	1.209 (2)	N4—O5	1.231 (2)
N2—C2	1.499 (2)	N4—C7	1.449 (3)
C9—C8	1.375 (3)	C7—C6	1.375 (3)
C9—N5	1.461 (2)	C7—C8	1.381 (3)
N3—O4	1.199 (2)	C6—C5	1.370 (3)
N3—O3	1.213 (2)	C6—H6	0.9300
N3—C2	1.504 (2)	C8—H8	0.9300
N5—O7	1.223 (2)	C5—H5	0.9300
			
C4—N1—C1	132.01 (15)	C2—C3—H3B	113.9
C4—N1—C3	124.20 (14)	N1—C3—H3A	113.9
C1—N1—C3	93.91 (13)	C2—C3—H3A	113.9
N1—C4—C5	117.53 (15)	H3B—C3—H3A	111.1
N1—C4—C9	126.61 (15)	O6—N4—O5	122.96 (19)
C5—C4—C9	115.86 (16)	O6—N4—C7	118.91 (19)
O1—N2—O2	125.62 (17)	O5—N4—C7	118.1 (2)
O1—N2—C2	116.82 (15)	C6—C7—C8	121.05 (18)
O2—N2—C2	117.57 (16)	C6—C7—N4	119.64 (18)
C8—C9—C4	121.83 (16)	C8—C7—N4	119.31 (18)
C8—C9—N5	115.41 (15)	C5—C6—C7	119.63 (17)
C4—C9—N5	122.54 (16)	C5—C6—H6	120.2
O4—N3—O3	125.76 (19)	C7—C6—H6	120.2
O4—N3—C2	115.52 (16)	N2—C2—N3	105.99 (14)
O3—N3—C2	118.72 (17)	N2—C2—C3	116.55 (14)
O7—N5—O8	123.01 (16)	N3—C2—C3	114.47 (15)
O7—N5—C9	118.51 (17)	N2—C2—C1	116.00 (15)
O8—N5—C9	118.39 (15)	N3—C2—C1	114.20 (14)
N1—C1—C2	88.22 (12)	C3—C2—C1	89.46 (13)
N1—C1—H1A	113.9	C9—C8—C7	119.30 (17)
C2—C1—H1A	113.9	C9—C8—H8	120.3
N1—C1—H1B	113.9	C7—C8—H8	120.4
C2—C1—H1B	113.9	C6—C5—C4	122.15 (17)
H1A—C1—H1B	111.1	C6—C5—H5	118.9
N1—C3—C2	88.23 (12)	C4—C5—H5	118.9
N1—C3—H3B	113.9		
			
C1—N1—C4—C5	−151.31 (18)	O1—N2—C2—C3	−140.71 (18)
C3—N1—C4—C5	−15.0 (3)	O2—N2—C2—C3	39.7 (2)
C1—N1—C4—C9	28.7 (3)	O1—N2—C2—C1	−37.3 (2)
C3—N1—C4—C9	165.05 (17)	O2—N2—C2—C1	143.13 (16)
N1—C4—C9—C8	−175.18 (17)	O4—N3—C2—N2	−178.93 (15)
C5—C4—C9—C8	4.9 (3)	O3—N3—C2—N2	1.3 (2)
N1—C4—C9—N5	10.4 (3)	O4—N3—C2—C3	51.2 (2)
C5—C4—C9—N5	−169.55 (16)	O3—N3—C2—C3	−128.56 (18)
C8—C9—N5—O7	23.2 (2)	O4—N3—C2—C1	−50.0 (2)
C4—C9—N5—O7	−162.06 (18)	O3—N3—C2—C1	130.29 (18)
C8—C9—N5—O8	−153.55 (17)	N1—C3—C2—N2	122.10 (16)
C4—C9—N5—O8	21.2 (2)	N1—C3—C2—N3	−113.44 (15)
C4—N1—C1—C2	148.25 (19)	N1—C3—C2—C1	3.06 (14)
C3—N1—C1—C2	3.19 (14)	N1—C1—C2—N2	−122.59 (15)
C4—N1—C3—C2	−152.23 (17)	N1—C1—C2—N3	113.68 (16)
C1—N1—C3—C2	−3.19 (14)	N1—C1—C2—C3	−3.07 (14)
O6—N4—C7—C6	175.89 (19)	C4—C9—C8—C7	−2.9 (3)
O5—N4—C7—C6	−3.2 (3)	N5—C9—C8—C7	171.93 (16)
O6—N4—C7—C8	−4.3 (3)	C6—C7—C8—C9	−1.1 (3)
O5—N4—C7—C8	176.56 (19)	N4—C7—C8—C9	179.16 (17)
C8—C7—C6—C5	2.7 (3)	C7—C6—C5—C4	−0.4 (3)
N4—C7—C6—C5	−177.53 (18)	N1—C4—C5—C6	176.83 (18)
O1—N2—C2—N3	90.61 (19)	C9—C4—C5—C6	−3.2 (3)
O2—N2—C2—N3	−88.97 (19)		
